# 70% Alcohol Versus Dry Cord Care in the Umbilical Cord Care: A Case–Control Study in Italy

**DOI:** 10.1097/MD.0000000000003207

**Published:** 2016-04-08

**Authors:** Rosanna Quattrin, Kim Iacobucci, Anna Lisa De Tina, Letizia Gallina, Carla Pittini, Silvio Brusaferro

**Affiliations:** From the Azienda Ospedaliero-Universitaria “Santa Maria della Misericordia” (RQ, ALDT, CP); School of Nursing, University of Udine (KI, LG); and Department of Medical and Biological Sciences, University of Udine (SB), Udine, Italy.

## Abstract

Recently the use of antibacterial agents to clean and dry the stump of the newborns’ umbilical cord (UC) after birth has been abandoned by many neonatal units in favor of dry cord care. Aim of this study was to compare the occurrence of adverse events (AEs) and time to cord separation among newborns treated with dry cord care versus 70% alcohol in an Italian Academic Hospital (AH).

From December 2014 to March 2015, 239 infants were born at the AH. The number of eligible infants was 200 and they were equally assigned to either case group (dry cord care) or control group (70% alcohol, standard procedure). Standard cord care consisted in 1 application of 70% alcohol at birth followed by other 2 times a day, while experimental dry cord care procedure was executed by the only application of a sterile gauze around the base of the UC at the 1st day of life and after the cord has been exposed to air off the diaper edge. The time to UC separation and any AEs such as local and systemic infections, hemorrhage, and granuloma formation were reported by mothers.

We found a significant difference in the mean cord separation time between the 2 groups (dry cord care: 10.1 days [standard deviation, SD = 4.0] vs 70% alcohol: 12.0 days [SD = 4.2]; *P* < 0.001), while no significant AEs resulted. Incidence rate of granuloma was 0.67 × 1000 days of life in dry cord care group.

Dry cord care is an easy, straight-forward, and safe method of handling the UC in healthy newborn infants born in a high-income hospital setting.

## INTRODUCTION

The umbilical cord (UC) which connects the baby and placenta in uterus (the womb) is made of blood vessels and connective tissue. It is covered by a membrane that is normally bathed in amniotic fluid. After birth, cutting the cord physically and symbolically separates the mother and her baby. The cord stump (CS) dries, falls off, and the wound heals.^1^

The cord usually separates between 5 and 15 days after birth. Before the separation, the remaining stump can be considered to be a healing wound and thus a possible route for infection through the vessels into the baby's blood stream.^1^

Soon after a normal delivery, the skin of the newborn baby including the CS is colonized mainly by nonpathogenic bacteria such as coagulase negative *Staphylococci* and *Diphtheroid* bacilli. Pathogenic bacteria such as *Coliforms* and *Streptococci* may also be present on the skin^2^ and can track up the CS causing infection.

In the developing countries, one-third of the deaths are caused by infections, mostly because of the delivery environment (generally the community and the houses). Cord infection may be localized to the UC (omphalitis) or, after its entry into the blood stream, it becomes systemic (e.g., neonatal sepsis).^2^ The most observed infections upon the CS and the abdominal surface are due to bacterial omphalitis with polymicrobic aetiology,^3^ but also to *Clostridium tetani*.^4^ The onset of the symptoms is usually observed between the 5th and the 9th day of life.

While there is a general agreement about the clean technique for cutting the cord using a sterile cutting instrument (blade or scissors) and regard to clean hands to avoid infection, there is less accord on what is the best care of the CS.^1^

Internationally, the World Health Organization (WHO) has advocated since 1998 for the use of dry UC care (keeping the cord clean without application of anything and leaving it exposed to air or loosely covered by a clean cloth, in case it becomes soiled it is only cleaned with water).^5^ Also the American Academy of Pediatrics considers no antiseptic treatment to be superior to any other^6^ and the guidelines from the German Association for Neonatology and Pediatric Intensive Care recommend clean care and keeping the UC dry.^7^

On the basis of a Cochrane review^1^ and other several studies,^8–12^ WHO recommends daily chlorhexidine (7.1% chlorhexidine digluconate aqueous solution or gel, delivering 4% chlorhexidine) application to the UC stump during the 1st week of life for newborns who are born at home in settings with high neonatal mortality (30 or more neonatal deaths per 1000 live births), while dry cord care for newborns born in health facilities and at home in low neonatal mortality settings. The use of chlorhexidine in these situations may be considered only to replace application of a harmful traditional substance, such as cow dung, to the CS. This is classified as a strong recommendation based on low to moderate quality evidence.^13^

Aim of this study was to compare the occurrence of all adverse events (AEs) and the cord separation time among newborns treated with dry cord care versus 70% alcohol in an Italian Academic Hospital (AH) to give a valid recommendation to clinicians.

## METHODS

Study subjects were recruited from December 2014 to March 2015 at Azienda Ospedaliero-Universitaria of Udine, a large Italian North Eastern AH, where about 1600 deliveries take place annually. The minimum sample size (N = 150) was calculated using the formula of estimating a single population (N = 1600) portion, taking 15% proportion of 5% margin of error and 95% confidence level.

Infants were considered eligible according to the inclusion criteria reported in Table 1 which are in line with inclusion criteria in other similar studies.^11,14^ After obtaining written consent by parents, subjects were assigned to control group if born in December 2014 and January 2015, and to case group if born in February 2015 and March 2015. Standard cord care consisted in 1 application of 70% alcohol at birth followed by other 2 times a day, while experimental dry cord care procedure was executed by the only application of a sterile gauze around the base of the UC on the 1st day of life to absorb bloody secretions and after the cord has been exposed to air off the diaper edge. Mothers were instructed to wash the area around CS carefully with water and dried when bathing the baby and to fold nappy and plastic under cord area, leaving cord exposed to air. Also parents were educated to observe and report any signs of infection, that is, redness, stickiness, or offensive odor.

**TABLE 1:**
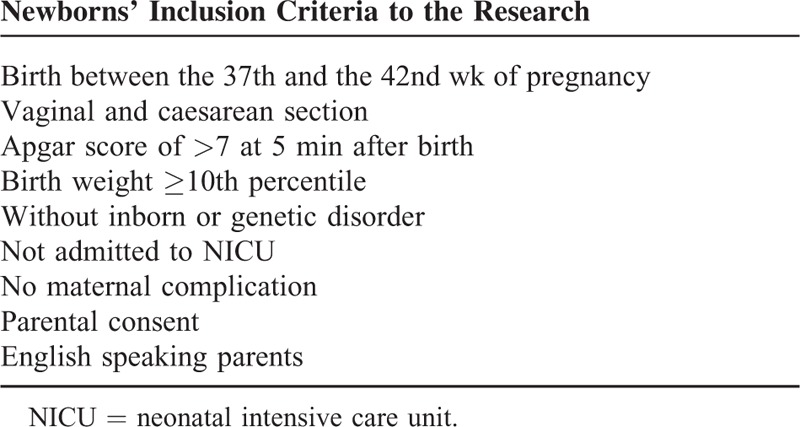
Criteria to Including Newborns in the Experimental Study

A researcher collected personal data and mother's and the newborn's medical history reported in medical records. Delivery (natural, caesarean section, and vacuum extractor), birth weight, sex, ethnic origin, hygiene and comfort after birth (immersion bath or sponging), administration of phototherapy, and type of breastfeeding at discharge were the considered variables.

A questionnaire (available on request) was administered by phone to mothers 1 month after childbirth to investigate: type of breastfeeding at home, occurrence of AEs and their treatments, cord separation time, changes in cord care procedures at home, admission to hospital, and access to outpatient care. Parents were given a calendar where daily they had to report any changes in the umbilical wound and the day of the stump fall.

Parents were asked to record signs of UC infection including pus and redness (inflammation), swelling (edema), or both, of the CS and skin at the base of the stump. Infections were categorized into 4 gradations (none, mild, moderate, and severe) according Mullany.^10^ Mild was defined as redness or swelling limited to the CS only; moderate was defined as <2 cm extension onto the abdominal skin at the base of the CS; and severe was defined as spreading noticeably (>2 cm) outward from the base of the stump. In case of systemic infection (also known as neonatal sepsis), the describing criteria were the admission diagnosis of the newborn in the pediatric unit, the contingent analysis, and the cultural exams. The study also detected the umbilical granuloma that is the most common umbilical abnormality in the neonate.^11^

Criterion to define CS fall was the complete detachment of the stump from the newborn's abdominal surface.

Ethical approval for this study was not needed, because dry cord care was already approved by the scientific community and because the study did not include the use of antiseptics or other substances.

Data collected were entered in an Excel spreadsheet and were analyzed using the statistical software SPSS, version 20. Pearson chi-squared test and Mann–Whitney test, for mean comparison, were used. Statistical significance was defined as *P* ≤ 0.05.

## RESULTS

Two hundred inborn healthy term infants were recruited from December 2014 to March 2015 distributed equally in experimental group (dry cord care) and control group (70% alcohol).

Table 2 shows the newborns’ characteristics in the 2 groups under study.

**TABLE 2:**
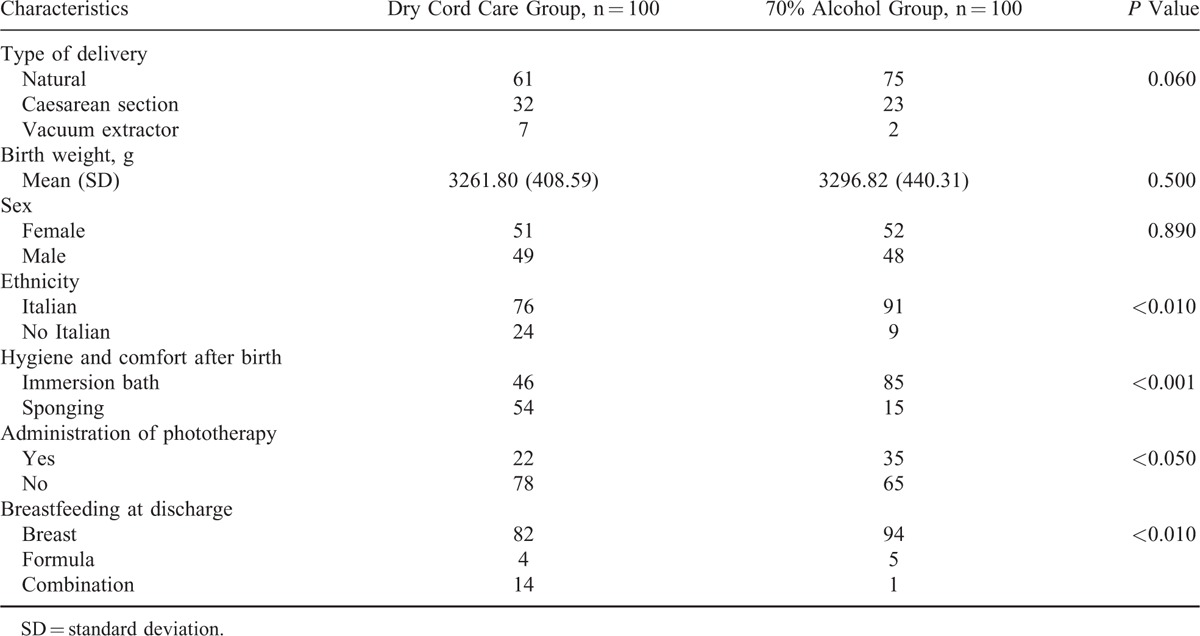
Baseline Characteristics of Subjects in the 2 Groups Under Study

Table 3 reports questions administered to mothers at home 1 month after childbirth: type of breastfeeding at home, occurrence of AEs and their treatments (admission in hospital or access to outpatient care), changes in cord care procedures at home, and cord separation time. The 2 cases of umbilical granuloma were treated with silver nitrate stick.

**TABLE 3:**
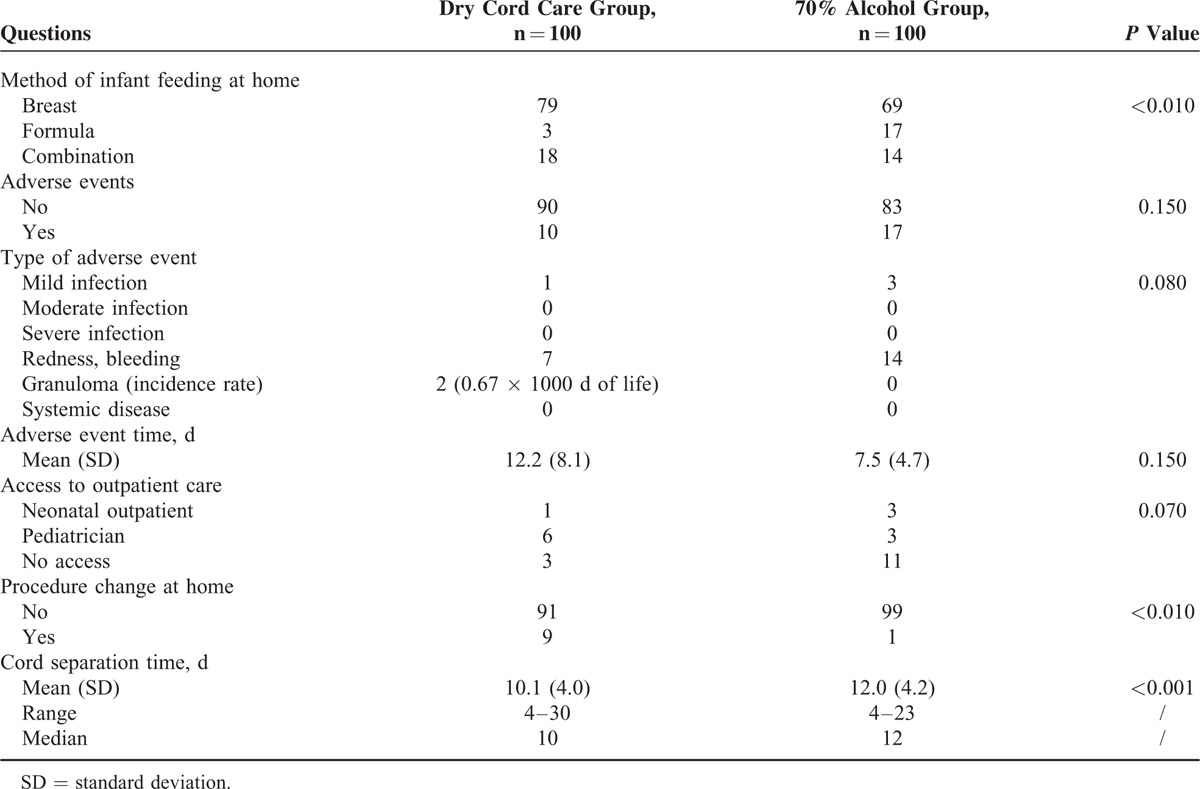
Questions Administered to Mothers at Home One Month After Childbirth, Distributed in the 2 Groups Under Study

Comparing means of UC separation time between group treating with phototherapy and one that was not undergone to phototherapy, no statistical differences resulted among the 2 groups, while considering only newborns treated with phototherapy a significant statistical differences emerged between case and control groups (9.9 days [standard deviation, SD = 3.7] in dry cord care vs 11.6 days [SD = 4.0] in 70% alcohol; *P* < 0.01).

## DISCUSSION

Even if in 2013 WHO recommended clean dry cord care for infants born in health facilities and at home in low neonatal mortality settings, healthcare working in neonatal unit and caregivers use different procedures to care the UC according their experiences and their preferences. Also in high-income countries, where mortality is very low, important outcomes in the 1st month^1^ of life regard to UC care must include more frequently AEs such as irritation, redness of the navel wall, weeping and bleeding of the navel, rarely infections like omphalitis, sepsis, and umbilical granuloma, and the time to separation of the UC stump.^11^

This case–control study compared 2 UC care procedures: dry cord care and 70% alcohol. Dry cord care is the procedure in which the umbilical stump is kept “clean and dry without applying anything” where anything stands for a dye, an antiseptic, or an antibiotic.^5^ In this study, a sterile gauze around the base of the UC was applied only on the 1st day of life to absorb bloody secretions. The research found no difference in occurrence of UC AEs in the 2 groups. It confirms data shown in other trials conducted in healthy term infants born in high-income or middle-income hospital setting.^2,15,16^

Although the study was open, treatment bias was not relevant. Blinding was impossible due to the 2 very different cord care procedures. Treatment bias related to parents was not relevant because all subjects born from December 2014 to January 2015 were assigned to standard procedure and all subjects born from February 2015 to March 2015 were handled with dry cord care. It follows that mothers admitted to the hospital in the same period received the same UC care for their infants.

Some variables collected in this study were distributed in different percentages among the 2 groups, such as ethnic origin, hygiene at birth, phototherapy treatments, breast-feeding but these characteristics did not influence the occurrence of UC complications. Regard to ethnic origin it is remarkable the fact that in dry cord care group there were more no Italian infants but at the same time few of their mothers changed UC care procedure at home, although the literature reported widespread potentially harmful traditional practices, including use of herbs mixed with cooking oil or water, especially in Africa and in Asia.^17,18^ About hygiene at birth, we did not find studies referring that immersion bath and sponging after birth had difference occurrences of cord infections and bacterial colonization.^19–21^ With respect to administration of phototherapy, the literature did not report more UC AEs in newborns undergoing to this treatment. Instead studies demonstrated that breast-feeding was significantly related to less incidence of cord infection^22,23^ but in our research it was difficult to draw a conclusion because at discharge the percentage of women that nursed was higher in case group than in control group, while at home it was the opposite.

In literature, the mean UC separation time ranged from 4 to 16 days depending on the intervention and study setting.^2–10^ Studies which applied nothing to the cord had mean separation times of about 9 days.^14,24,25^ Meta-analysis of 4 studies with alcohol as the comparator showed a trend toward cord separation being significantly prolonged in the alcohol group but there was no significant difference in cord separation,^1^ while the present study showed a statistical difference between the 2 groups: in DCC the mean UC separation time was about 2 days before of control group 1 (10 days vs 12 days). The clinical impact of delays of cord separation is unknown, but it has social and cost implications: delay makes mothers anxious, and it increases the number of domiciliary midwife visits to the home.^26^ It follows that health workers and families prefer more rapid cord separation.^2^

An other consideration consists in the fact that in our study, phototherapy did not influence UC separation time in both case and control groups differently from literature reported that phototherapy delayed cord separation.^27^

This study detected also the occurrence of granuloma. It is an over growing tissue during the healing process of the belly button, usually occurs in reaction to a mild infection. It is not a congenital abnormality but represents continuing inflammation of granulation tissue, that has not yet epithelialized.^28^ The umbilical granuloma is the most common umbilical problem in infants but, to our knowledge, there are not studies that reported incidence rate in terms of its occurrence per 1000 days from childbirth but only the incidence percentages in 1 study of Kapellen et al^11^ (12.8% in clorexidine group and 11.7% in dry cord care group). A significant finding of the present study was the calculation of incidence rate of granuloma equal to 0.67 × 1000 days of life in dry cord care group.

The study had 3 limitations. First, it was conducted on babies and their mothers who were only eligible for selection criteria, and it may not be generalized to other cultures or countries. The second limitation is related to difficulties of standardized procedures in collecting the culture samples from UC in home setting. Therefore, it was not possible to provide the determination of microorganisms responsible of detected complication. Third, data on UC complications and treatments were obtained by telephone interview of mothers and not through direct observation by healthcare professionals. Before hospital discharge, parents were of course instructed to look for the warnings signs of UC complications and to contact their healthcare provider if in doubt.

## CONCLUSION

This case–control study compared 2 different procedures for the UC care in infants born in a high-come hospital: dry cord care versus 70% alcohol. No statistically significant differences between the 2 UC care practices resulted regard to the occurrence of UC AEs (local infection, systemic disease, granuloma, bleeding, etc.), while time of UC separation was significant shorter in dry cord care group. Dry cord care is an easy, straight-forward, and safe method of handling the UC in healthy newborn infants born in a high-income hospital setting.
